# Selective anticancer activity of *Vachellia nilotica* fruit extract: integrated phytochemistry with antioxidant, antimicrobial, and cancer cell targeting

**DOI:** 10.3389/fnut.2026.1799072

**Published:** 2026-04-07

**Authors:** Essam Y. Abdul-Hafeez, Omer H. M. Ibrahim, Hani A. Alfheeaid, Hassan Barakat

**Affiliations:** 1 Department of Plant Production, College of Agriculture and Food, Qassim University, Buraydah, Qassim, Saudi Arabia; 2 Department of Agriculture, Faculty of Environmental Sciences, King Abdulaziz University, Jeddah, Saudi Arabia; 3 Floriculture Department, Faculty of Agriculture, Assiut University, Assiut, Egypt; 4 Department of Food Science and Human Nutrition, College of Agriculture and Food, Qassim University, Buraydah, Qassim, Saudi Arabia

**Keywords:** antimicrobial activity, antioxidant activity, cancer cell lines, cytotoxicity, *Vachellia nilotica*, food supply

## Abstract

**Introduction:**

*Vachellia nilotica* has long been employed in traditional medicine for its therapeutic value. This study aimed to assess the phytochemical composition, antioxidant potential, antimicrobial efficacy, and *in vitro* anticancer activity of its methanolic fruit extract, thereby validating its ethnopharmacological significance.

**Methods:**

The methanolic extract was subjected to preliminary phytochemical screening for key secondary metabolites. Total phenolic and flavonoid contents were quantified using Folin–Ciocalteu and aluminum chloride colorimetric methods, respectively. The antioxidant capacity was evaluated through the DPPH free radical scavenging assay. Antibacterial and antifungal activities were determined by the agar well diffusion method against pathogenic strains. The cytotoxic and selectivity profiles were assessed using MTT assays on human cancer cell lines (MCF-7, PC-3, Caco-2) and normal Vero cells, with doxorubicin serving as a positive control.

**Results:**

Qualitative screening revealed the presence of tannins, alkaloids, glycosides, flavonoids, and terpenoids. The extract exhibited strong antioxidant potential with an IC_50_ of 31.77 μg mL^−1^, supported by high total phenolic (419.45 mg GAE g^−1^) and flavonoid (245.48 mg QE g^−1^) contents. Notable antibacterial effects were observed against both Gram-positive and Gram-negative bacteria, with maximum inhibition zones of 23.00 mm for *Acinetobacter johnsonii* and 21.00 mm for *Serratia marcescens* at 1000 μg mL^−1^. Pronounced antifungal inhibition was recorded against *Rhizoctonia solani*, *Penicillium italicum*, and *Fusarium oxysporum*. The extract exhibited moderate and selective cytotoxicity toward cancer cells, reducing viability to below 5% at 500–1000 μg mL^−1^, while maintaining significantly higher viability in normal Vero cells compared with doxorubicin.

**Discussion:**

The findings underscore the potent antioxidant, antimicrobial, and selective anticancer properties of *V. nilotica* fruit extract. These bioactivities substantiate its traditional medicinal applications and highlight its potential as a source of bioactive compounds for therapeutic development. Further *in vivo* and mechanistic studies are warranted to elucidate its pharmacological potential and clinical relevance.

## 1 Introduction

The World Health Organization (WHO) reports that herbal medicine constitutes the primary healthcare approach for approximately 80% of the world’s population, mainly owing to several key advantages: lower cost and widespread availability, particularly in resource-limited settings; higher patient compliance and satisfaction; and reduced incidence of adverse reactions compared to conventional synthetic pharmaceuticals (1). Previous studies have validated the use of medicinal herbs as alternatives for managing infections, diabetes, cancer, and various other diseases, ranging from minor to moderate conditions (2–5).

*Vachellia nilotica* (L.) P. J. H. Hurter & Mabb., synonymously known as *Acacia nilotica* (L.) Willd. ex Delile, is classified within the family Fabaceae (6, 7). It exhibits multiple regional nomenclatures, including the talh tree, gard, and gum arabic tree. It is naturally distributed across numerous geographic regions, particularly in Saudi Arabia, India, Egypt, and Sri Lanka (8). Laboratory studies and epidemiological evidence support the role of chemoprevention in reducing the risk of cancer in humans (9, 10). Numerous plant-derived dietary constituents have demonstrated chemopreventive effects by inhibiting, suppressing, or reversing carcinogenesis induced by various environmental factors. *V. nilotica* (L.) exhibits notable antimutagenic properties, particularly against indirect mutagens (11). Various anatomical components of *V. nilotica*, namely, fruit, stem bark, and mature pods, have been employed in traditional medicine as therapeutic remedies. These plant parts function as tonic and astringent agents and have been utilized to manage respiratory infections, such as colds, bronchitis, and pneumonia, as well as gastrointestinal disorders, including dysentery (12). Several studies have verified its antimicrobial activity (8, 12, 13). These plant parts exhibit substantial enrichment in phenolic compounds, particularly condensed tannins and phlobatannins. Scientific literature documents that extracts derived from this plant exhibit diverse pharmacological activities, encompassing antimicrobial efficacy, anticancer properties, antimutagenic potential, antipyretic effects, anti-inflammatory action, anti-ulcer benefits, antihypertensive capacity, antispasmodic activity, and antioxidant properties (8, 14–18).

The fruits of *V. nilotica* contain diverse phytochemical constituents that substantially contribute to their medicinal efficacy. Among the prominent bioactive compounds, tannins are present in elevated concentrations and have traditionally been employed for their anti-filarial therapeutic properties. Previous phytochemical investigations of *V. nilotica* fruits have reported the presence of several galloylated catechin derivatives, including (+)-catechin-3-O-gallate, (−)-epicatechin-3-O-gallate, (+)-gallocatechin, (−)-epigallocatechin, and (−)-epigallocatechin-3-O-gallate (19). These catechin derivatives have potent anthelmintic potential. In addition to tannins, *V. nilotica* fruits harbor flavonoid and carbohydrate constituents. Conversely, previous studies have reported the absence of alkaloids in pod extracts; however, our analysis of the fruit extract identified alkaloids, suggesting tissue-specific variations in the phytochemical composition (20). This aligns with the broader phytochemical profile of *V. nilotica*, in which other parts of the plant have been found to contain phenolics, such as gallic acid and catechin derivatives, contributing to its extensive use in traditional medicine (21). Overall, the phytochemicals in *V. nilotica* fruits and other plant parts support their use in the treatment of various health conditions, underscoring their potential pharmacological and medicinal applications.

Bacterial infections pose significant clinical challenges in both acute and chronic cases. Appropriate diagnosis before treatment is essential to reduce the risk of antibiotic resistance (22). Despite ongoing innovations in antimicrobial therapeutics, the escalating prevalence of antimicrobial resistance poses a significant public health challenge (23). Resistance evolves under selective pressure created by antimicrobial use and is emerging at a pace that outstrips the introduction of new drugs. Effective antimicrobial stewardship, prudent drug use, alternative therapies, and infection prevention strategies are urgently required to slow this trend (24).

The literature indicates that methanolic extracts of *V. nilotica* fruit exhibit higher phenolic content and antioxidant activity than extracts obtained with other solvents. Consequently, this study aimed to (1) characterize the antibacterial properties of *V. nilotica* fruit extract and establish its estimated minimum inhibitory concentration (MIC-estimate), (2) evaluate its cytotoxic effects on MCF-7, PC-3, and Caco-2 cancer cells and non-transformed Vero cells, and (3) quantify its antioxidant capacity.

## 2 Materials and methods

### 2.1 Sample collection and condensation process

Dried fruit specimens of *V. nilotica* (L.) P. J. H. Hurter & Mabb. (synonym: *Acacia nilotica* (L.) Willd. ex Delile were procured from the local herbs market at Buraydah, Qassim Region, Kingdom of Saudi Arabia. The fruits were pulverized and thoroughly rinsed with water. A 100 g aliquot of resultant fruit pulp was subsequently immersed in 1000 mL of 80% methanolic solution and subjected to continuous agitation for 48 h at ambient temperature (25 ± 2 °C). Following the extraction period, the mixture was filtered, and the solvent was removed by rotary evaporation at 45 °C. The concentrated extract was preserved in a sealed container at 4 °C for subsequent analyses. The extraction process yielded 42.0% recovery, corresponding to a final extract mass of 42.0 g obtained from the initial 100 g sample.

### 2.2 Preliminary phytochemical screening

Phytochemical screening was performed using an established standard methodology with minor modifications based on the protocols described by Farnsworth et al. (25), Harborne (26), and Sofowora (27).

#### 2.2.1 Phenolic compounds and tannins (ferric chloride test)

Phenolic compounds were detected using the ferric chloride assay. A 50 mg extract sample was dissolved in 5 mL of distilled water, and a small volume of neutral ferric chloride solution was added. The development of dark green coloration confirmed the presence of phenolic compounds (26).

#### 2.2.2 Alkaloids (Mayer’s test)

Alkaloid identification was performed using Mayer’s reagent precipitation assay. Two drops of Mayer’s reagent were carefully applied to the inner walls of a test tube containing a plant extract sample (several milliliters). The development of a creamy white or pale yellow precipitate indicated a positive alkaloid result (28).

#### 2.2.3 Glycosides (Borntrager’s test)

Borntrager’s test was used to identify the presence of glycosides. In this procedure, 2 mL of the filtered hydrolysate was combined with 3 mL of chloroform and subsequently agitated. Subsequently, the chloroform layer was extracted and replaced with a 10% ammonia solution. The appearance of a pinkish hue indicated the presence of glycosides (27).

#### 2.2.4 Flavonoids (aluminum chloride test)

Flavonoid compounds were identified through aluminum chloride-based chemical analysis. The analytical procedure involved mixing 3 mL of the plant extract with a 1% AlCl_3_ solution. The appearance of yellow coloration indicated the presence of positive flavonoid compounds (29).

#### 2.2.5 Terpenoid test (Salkowski test)

Terpenoid detection was performed using the Salkowski colorimetric assay. Next, 5 mL of the extract sample was combined with 2 mL of chloroform, and 3 mL of concentrated sulfuric acid was carefully added to establish a distinct stratified layer. The appearance of a reddish-brown coloration at the interface indicated the presence of terpenoids (26).

#### 2.2.6 Saponins (frothing test)

Saponin detection was performed using a frothing assay. Next, 50 mg of the extract sample was dissolved in distilled water to reach a final volume of 20 mL. The mixture was vigorously agitated in a graduated cylinder for 15 min. The formation of a foam layer with a height of 2 cm indicated the presence of saponins (27).

### 2.3 *In vitro* antioxidant activity (DPPH˙ radical scavenging assay)

The antioxidant activity of the methanolic fruit extract was evaluated using the DPPH radical scavenging method, as described by Chang et al. (29). The experiment involved mixing 1 mL of 0.1 mM DPPH in methanol with 1 mL of the extract samples at multiple concentrations (20–120 μg/mL) and incubating the solution in the dark for 30 min. A blank control was established by combining 1 mL of methanol with 1 mL of DPPH solution. Absorbance measurements were performed at 517 nm using UV–Vis spectrophotometry with ascorbic acid as the reference standard. The percentage of radical inhibition was determined using Eq. 1:


$$\%\text{of DPPH radical inhibition}=(\frac{\mathrm{Abs}.\text{control}-\mathrm{Abs}.\text{sample}\;}{\mathrm{Abs}.\text{control}})\times 100$$ (1)

### 2.4 Total phenolic content determination

The total phenolic content was quantified using a spectrophotometric assay adapted from the Folin–Ciocalteu methodology (30), with minor procedural adjustments. An aliquot (0.1 mL) of the methanolic extract was transferred to a tube, followed by the addition of 0.2 mL of 10% Folin–Ciocalteu reagent (Panreac, Barcelona, Spain) dissolved in water (v/v) to each standard and sample tube. The resulting mixture was vortexed for 10 s, sealed, and incubated at ambient temperature for 30 min. Subsequently, 0.8 mL of 700 mM aqueous sodium carbonate (Na_2_CO_3_) was added, and the mixture was vortexed again, sealed, and maintained at room temperature for 2 h. A precisely measured 0.25 mL aliquot of each sample was deposited in triplicate into a 96-well microplate. During phenolic oxidation, the phosphomolybdic and phosphotungstic acid components of the Folin–Ciocalteu reagent undergo reduction, producing blue-colored molybdenum and tungsten oxide species. Absorbance readings were recorded at a wavelength of 735 nm against a blank sample (in triplicate). The total phenolic content was quantified as gallic acid equivalents using a calibration curve, and results were reported as mg gallic acid per g. The calculation employed the regression equation, *y* = 0.0044*x* + 0.0384. It was expressed as mg gallic acid equivalent (GAE) g^−1^ extract using the formula *T* = CV/M, where *T* is the total phenolic content (mg GAE g^−1^ extract), C is the concentration of gallic acid (μg mL^−1^) from the calibration curve, *V* is the volume of the extract, and *M* is the mass of the extract.

### 2.5 Total flavonoid content determination

Flavonoid quantification in the *V. nilotica* extract was performed using spectrophotometric analysis and reported as quercetin equivalents, employing a modified aluminum chloride colorimetric assay (29). The analytical procedure involved combining 0.5 mL of the extract solution (1,000 μg mL^−1^) with 1.5 mL of ethanol, 0.1 mL of 1 M potassium acetate (w/v), 0.2 mL of 10% aluminum chloride (w/v), and 2.7 mL of distilled water. The resulting mixture was held at ambient temperature (28 °C) for 30 min, after which the absorbance was quantified at 430 nm using a microplate spectrophotometer. Distilled water served as the blank control in place of the 10% aluminum chloride reagent. A calibration curve was established through linear regression analysis, with quercetin concentrations plotted on the x-axis and corresponding absorbance values on the y-axis. The quercetin-derived calibration equation (*y* = 0.0055*x* − 0.0258) was utilized, where y represents the sample absorbance and *x* denotes the sample concentration (range: 3–15 μg mL^−1^, *R* = 0.9944).

### 2.6 Antibacterial activity

#### 2.6.1 Bacterial strains

*Bacillus subtilis*, a gram-positive bacterium, and *Erwinia carotovora* subsp. *atroseptica,* a gram-negative bacterium, were isolated from soil and potato fields. The identification of these isolates was based on their morphological, biochemical, and physiological characteristics (31–34), and detailed diagnostic features are provided in Supplementary Table S1. Additional gram-negative bacteria were obtained from strains deposited at the Department of Plant Pathology, Assiut University, Egypt: *Escherichia coli* (DH5α), *Acinetobacter johnsonii 6,005, Agrobacterium tumefaciens 614,* and *Serratia marcescens 2039*.

#### 2.6.2 Antibacterial activity test

Extract sterilization was achieved using 0.20-μm polyethersulfone (PES) syringe filtration. The agar well diffusion technique was employed for antimicrobial evaluation (35–37). Bacterial strains were cultivated in nutrient broth at 28 ± 1 °C for 12 h to achieve a concentration of 10^6^ CFU mL^−1^. *E. coli* was cultured in LB medium, and cell suspensions (150 μL) were homogeneously distributed on agar plates. Inoculation wells (6 mm diameter) were filled with 100 μL of crude extract and incubated for 24 h at 28 ± 1 °C. Ampicillin sodium salt (250 mg L^−1^) was used as the positive control compound. Bacterial susceptibility was indicated by inhibition zones ≥ 6 mm in diameter. Inhibition was determined using Eq. 2. The minimum inhibitory concentration (MIC-estimate; determined from the agar diffusion assay) was defined as the lowest extract concentration that caused ≥50% inhibition.


$$\%\text{Growth inhibition}=(\frac{\text{Zone}\;\text{control}-\text{Zone}\;\text{treatment}}{\text{Zone}\;\text{control}})\times 100$$ (2)

### 2.7 Antifungal activity

The antifungal properties of *V. nilotica* fruit extract were evaluated against three fungal isolates deposited at the Department of Plant Pathology, Assiut University, Egypt: *Rhizoctonia solani* 301, *Penicillium italicum* 309, and *Fusarium oxysporum* 389, as reported by Abdel-Hafez et al. (38). The extract was tested at varying concentrations (15.6–1,000 μg mL^−1^) incorporated into potato dextrose agar medium with hymexazole (1,000 μg mL^−1^) as the positive control. Hymexazole (1,000 μg/mL) served as the positive control because of its established efficacy against these plant-pathogenic fungi. Each plate received a 2 mm fungal culture disk at its center and was incubated for 10 days at 28 ± 1 °C. The percentage of mycelial inhibition was calculated using Eq. 3:


$$\begin{array}{l} \%\text{Mycelial Growth Inhibition}\\ =(\frac{\text{Diameter}\;\text{control}-\text{Diameter}\;\text{treatment}}{\text{Diameter}\;\text{control}})\times 100 \end{array}$$ (3)

### 2.8 Anticancer activity

#### 2.8.1 Cell cultures

The cell lines employed in the current study were obtained from the American Type Culture Collection (ATCC, Manassas, VA, USA). They included three human cancer cell lines: prostate (PC-3, accession number: ATCC CRL-1435), breast (MCF-7, accession number: ATCC HTB-22), and colorectal adenocarcinoma (Caco-2, accession number: ATCC ATB-37), as well as the normal Vero cell line (accession number: ATCC CCL-81). RPMI medium was used to grow cells with the addition of 10% fetal bovine serum, 100 U mL^−1^ penicillin G, and 100 mg mL^−1^ streptomycin sulfate. The cultured cells were incubated at 37 °C in a CO_2_ incubator and harvested using trypsin (0.25%) and EDTA-2Na (0.025%) in PBS.

#### 2.8.2 MTT assay

Cytotoxicity was evaluated using the MTT cell viability assay (4, 5). A density of 1 × 10^5^ cells mL^−1^ was seeded into 96-well microplates and cultured for 24 h. Dose–response studies were initiated by adding extract concentrations ranging from 31.25 to 1,000 μg mL^−1^, along with doxorubicin (positive control), which was tested at concentrations ranging from 31.25 to 1,000 μg mL^−1^, and incubating for 24 h. MTT reagent (20 μL of 5 mg mL^−1^) was added, incubated in the dark for 4 h, removed, and replaced with DMSO (200 μL) for 30 min. Absorbance was quantified at 560 nm. Cytotoxicity and selectivity index (SI) were calculated using (Eqs. 4 and 5):


$$\%\text{Cytotoxicity}=(\frac{\mathrm{Abs}.\text{control}-\mathrm{Abs}.\text{sample}}{\mathrm{Abs}.\text{control}})\times 100$$ (4)

$$\text{Selective Index}\;(\mathrm{SI})=(\frac{{\mathrm{IC}}_{50}\;\text{normal cells}}{{\mathrm{IC}}_{50}\;\text{cancer cells}})$$ (5)

An SI value exceeding 2 is typically considered indicative of selective cytotoxicity against cancer cells.

### 2.9 Statistical analysis

Statistical analyses were performed using Statistix software (version 8.1; Analytical Software, Tallahassee, FL, USA). Differences among multiple groups were evaluated using one-way analysis of variance (ANOVA), whereas comparisons between two groups were performed using unpaired *t*-tests. The least significant difference (LSD) test was used to compare means at the *p* ≤ 0.05 significance level. Results are presented as mean ± standard deviation (SD) derived from at least three independent experimental replicates (39).

## 3 Results

### 3.1 Phytochemical screening

**Figure 1:**
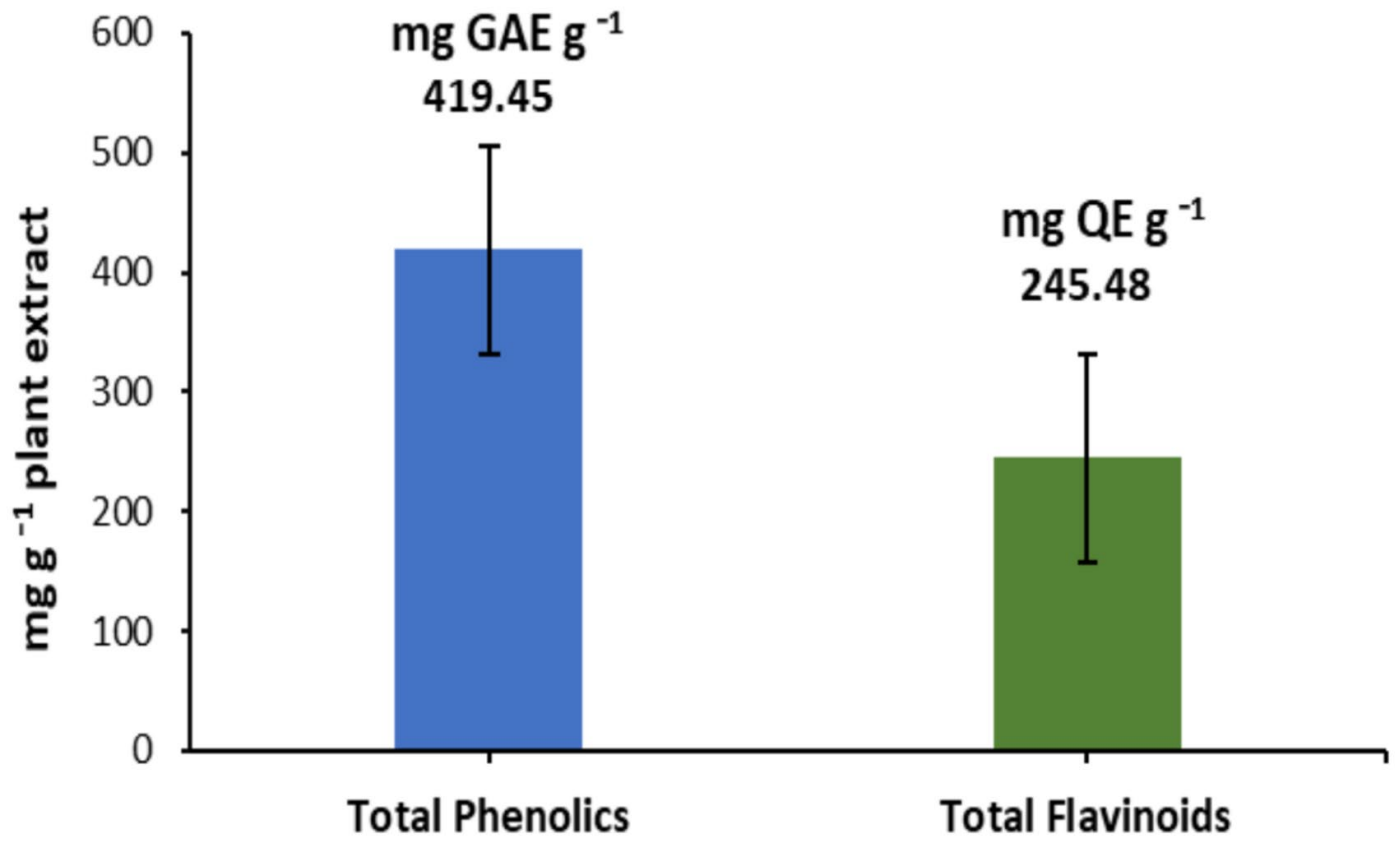
Total phenolic and flavonoid composition of *V. nilotica* extract. GAE, Gallic acid equivalent; QE, quercetin equivalent. The vertical bars above and within the columns represent standard deviation (*n* = 3).

Table 1 summarizes the detailed phytochemical composition of the 80% methanolic fruit extract derived from *V. nilotica*. Analytical findings demonstrated the presence of multiple bioactive constituents, including tannins, alkaloids, glycosides, flavonoids, terpenoids, and saponins. This extensive phytochemical repertoire indicates substantial potential for diverse pharmacological effects and therapeutic applications. Quantitative assessment further substantiated the bioactive potential of the extract, establishing elevated levels of total phenolic compounds (419.45 mg GAE g^−1^ extract) and flavonoid constituents (245.48 mg QE g^−1^ extract), as illustrated in Figure 1. These quantitative values underscore the extract’s status as a significant reservoir of phenolic and flavonoid compounds, which are likely responsible for the observed antioxidant properties.

**Table 1:** Preliminary phytochemical profiling and bioactive compound identification in *V. nilotica* fruit extract.

Phytochemical	Method/Reagent	Methanolic (80%) extract
Tannins	Ferric chloride test	+
Alkaloids	Mayer’s test	+
Glycosides	Borntrager’s test	+
Flavonoids	Aluminum chloride	+
Terpenoids	Salkowski test	+
Saponins	Frothing test	+

(+) indicates presence and (−) indicates absence.

### 3.2 *In vitro* antioxidant activity (DPPH˙ radical scavenging activity)

The antioxidant capacity of the methanolic *V. nilotica* fruit extract was determined using DPPH radical scavenging methodology. At 1000 μg mL^−1^, the extract exhibited 87.84% radical scavenging efficiency, with an IC_50_ value of 31.77 μg mL^−1^ (Table 2). The extract effectively converted stable DPPH radicals (purple) to the reduced form 1,1-diphenyl-2-picrylhydrazine (colorless), demonstrating concentration-dependent antioxidant activity. A comparison with the reference standard ascorbic acid (IC_50_ = 2.88 μg mL^−1^) provided context for the extract’s radical-scavenging potential.

**Table 2:** Antioxidant activity of the methanolic *V. nilotica* fruit extract measured via DPPH radical scavenging.

Dilutions (μg mL^−1^)	% Inhibition ±SD
31.25	48.04 ± 0.002
62.5	82.75 ± 0.001
125	87.06 ± 0.001
250	87.25 ± 0.003
500	87.65 ± 0.001
1,000	87.84 ± 0.002
IC_50_ (μg mL^−1^)	31.77

Results are reported as mean ± standard deviation based on triplicate determinations (*n* = 3).

### 3.3 Antimicrobial activity

The methanolic fruit extract of *V. nilotica* exhibited concentration-dependent antibacterial activity (Table 3). The largest inhibition zones were observed against *A. johnsonii* (23.00 mm) and *S. marcescens* (21.00 mm) at a concentration of 1,000 μg mL^−1^. However, the activity was lower than that of the standard ampicillin. In contrast, *E. coli* showed the least sensitivity, with only moderate inhibition at higher concentrations. Similarly, the extract exhibited notable antifungal activity (Table 4). The mycelial growth of *R. solani*, *P. italicum*, and *F. oxysporum* was significantly decreased in a concentration-dependent manner, although the inhibitory effect was less pronounced than that of hymexazol.

**Table 3:** Antimicrobial efficacy of the methanolic *V. nilotica* fruit extract against a panel of gram-positive and gram-negative bacterial species, quantified as zones of growth inhibition diameter (mm).

Extract Concentrations (μg mL^−1^)	Inhibition zone (mm)					
	*B. subtilis*	*E. carotovora*	*E. coli*	*S. marcescens*	*A. johnsonii*	*A. tumefaciens*
31.25	6.00ᵍ	6.00ᵍ	6.00ᶠ	6.00ᵍ	6.00ᶠ	6.00ᶠ
62.5	9.33ᶠ	6.00ᵍ	6.00ᶠ	7.67ᶠ	8.00ᵉ	8.00ᵉ
125	11.33ᵉ	6.00ᵍ	6.00ᶠ	12.33ᵉ	12.67ᵈ	14.00ᵈ
250	14.67ᵈ	11.00ᶠ	6.00ᶠ	15.33ᵈ	17.00ᶜ	16.33ᶜ
500	18.33ᶜ	13.00ᵉ	9.67ᵉ	18.00ᶜ	20.33ᵇ	19.33ᵇ
1,000	20.33ᵇ	16.00ᵈ	12.33ᵈ	21.00ᵇ	23.00ᵃ	21.33ᵃ
Negative control	6.00ᵍ	6.00ᵍ	6.00ᶠ	6.00ᵍ	6.00ᶠ	6.00ᶠ
Ampicillin (250 μg mL^−1^)	34.67ᵃ	34.00ᵃ	33.00ᵃ	41.33ᵃ	38.33ᵃ	37.00ᵃ
MIC-estimate (μg mL^−1^)	62.5	250	500	62.5	62.5	62.5

MIC-estimate: The minimum inhibitory concentration (MIC) estimated from an agar diffusion assay is the lowest concentration that causes ≥ 50% inhibition. Different superscript letters indicate significant differences (*p* ≤ 0.05, ANOVA with LSD post-hoc test).

**Table 4:** Antifungal efficacy of the methanolic *V. nilotica* fruit extract against selected plant-pathogenic fungal species, quantified as the diameter (mm) of the mycelial growth suppression.

Extract concentrations (μg mL^−1^)	Mycelial growth (mm)		
	*R. solani*	*P. italicum*	*F. oxysporum*
31.25	6.30ᶜ	4.33ᵈ	4.97ᶜ
62.5	5.40ᵈ	4.10ᵈ	4.67ᶜ
125	5.43ᵈ	5.37ᶜ	4.50ᶜ
250	4.87ᵉ	4.97ᶜ	4.30ᵈ
500	4.37ᶠ	4.83ᶜ	4.27ᵈ
1,000	4.13ᶠ	4.33ᵈ	3.87ᵉ
Negative control	9.00ᵃ	9.00ᵃ	9.00ᵃ
Hymexazol (1,000 μg mL^−1^)	1.83ᵍ	2.87ᵉ	1.63ᶠ
MIC-estimate (μg mL^−1^) ≥50% inhibition	500	62.5	125

Different superscript letters indicate significant differences (*p* ≤ 0.05, ANOVA with LSD post-hoc test.

### 3.4 *In vitro* cytotoxic activity

**Figure 2:**
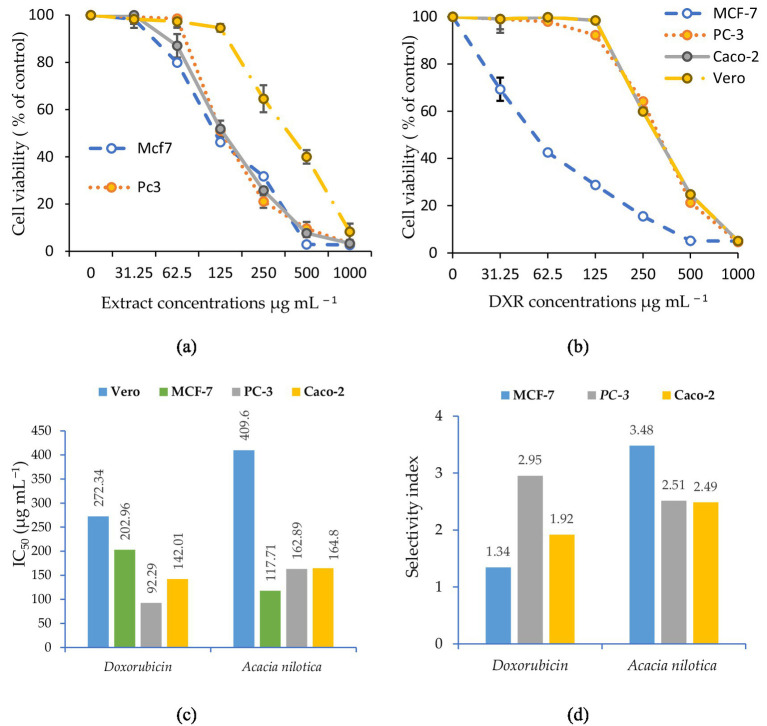
Comparative anticancer efficacy of *V. nilotica* methanolic fruit extract and doxorubicin across MCF-7, PC-3, Caco-2, and normal Vero cell lines: **(a)** Cytotoxic dose–response of the extract, reported as mean (*n* = 3) ± SD; **(b)** cytotoxic dose–response of doxorubicin, reported as mean (*n* = 3) ± SD; **(c)** half-maximal inhibitory concentration (IC_50_, μg mL^−1^) for both the extract and doxorubicin; and **(d)** therapeutic selectivity index (SI) of the extract.

The methanolic fruit extract of *V. nilotica* reduced the viability of MCF-7, PC-3, and Caco-2 cells in a concentration-dependent manner, with the most pronounced effect observed in MCF-7 cells (Figure 2). At higher concentrations, cell viability decreased sharply, reaching below 5% at 500–1000 μg mL^−1^ in all three cancer cell lines tested. In contrast, Vero cells showed comparatively higher survival at all concentrations, with 40.0% viability at 500 μg mL^−1^ and 8.3% viability at 1000 μg mL^−1^. Compared with doxorubicin (DXR), the standard reference drug, the extract exhibited lower potency but greater selectivity. DXR reduced cancer cell viability at much lower concentrations; however, it also caused marked cytotoxicity in Vero cells, particularly at concentrations above 250 μg mL^−1^. This difference indicates that although the extract is less potent than DXR, it exerts its effects with comparatively reduced toxicity in non-malignant cells.

### 3.5 Correlation heatmap between bacterial inhibition and antioxidant activity

**Figure 3:**
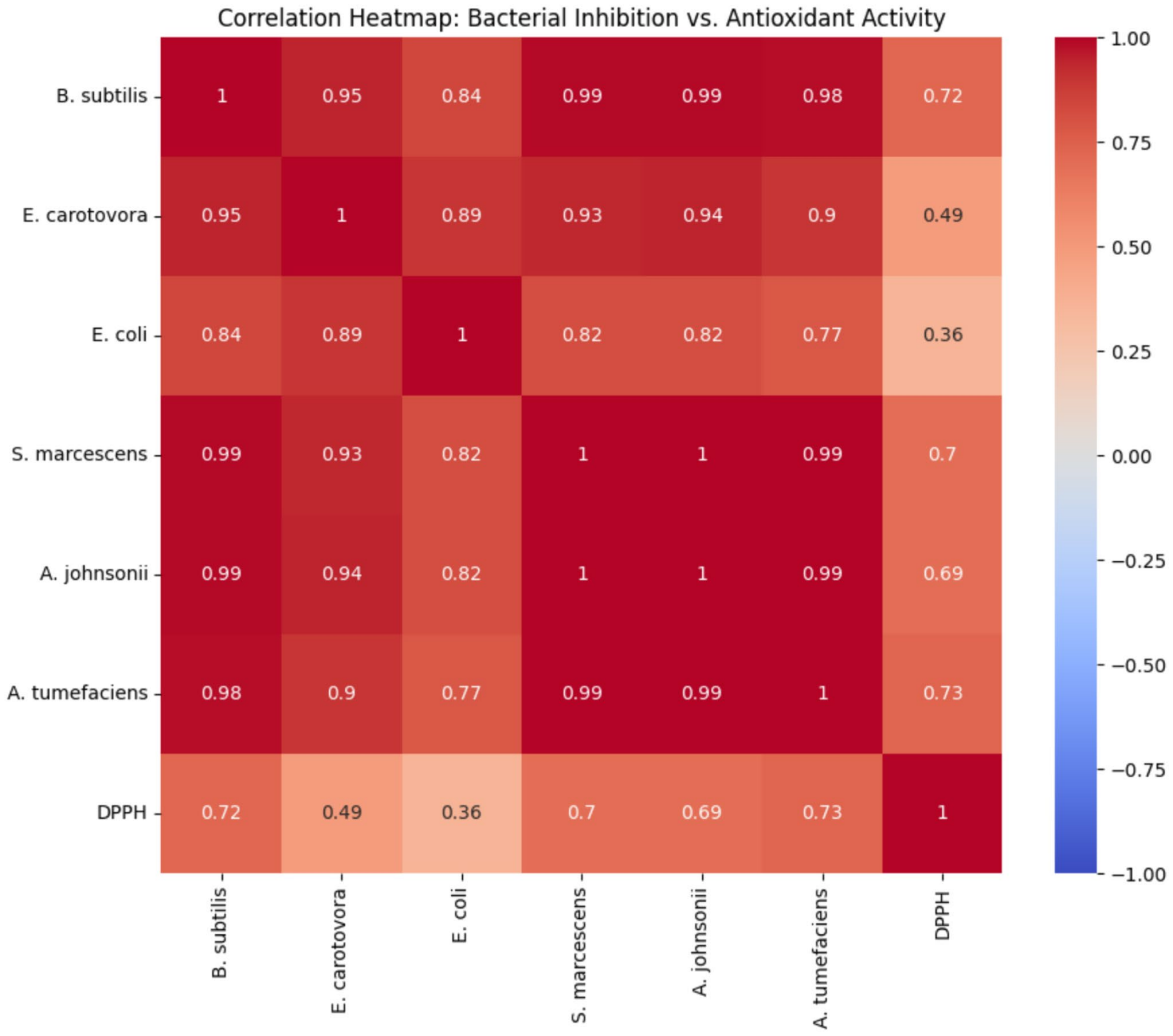
Correlation heatmap between bacterial inhibition and antioxidant activity under different extract concentrations.

The correlation heatmap (Figure 3) demonstrates the relationship between antioxidant activity and bacterial inhibition across the six bacterial strains at different extract concentrations. Antioxidant activity significantly increased from 48.04% at 31.25 μg mL^−1^ to 87.84% at 1000 μg mL^−1^, indicating the strong radical-scavenging potential of the extract. Correlation analysis revealed that antioxidant activity was strongly positively associated with the inhibition of *A. johnsonii* (*r* = 0.98, *p* < 0.001), *A. tumefaciens* (*r* = 0.98, *p* < 0.001), and *B. subtilis* (*r* = 0.93, *p* < 0.001). Moderate correlations were observed for *S. marcescens* (*r* = 0.95, *p* < 0.001) and *E. coli* (*r* = 0.95, *p* < 0.001), whereas *E. carotovora* displayed a slightly lower correlation (*r* = 0.96, *p* < 0.001). These results indicate that as the antioxidant capacity of the extract increased with concentration, the antibacterial activity generally increased as well, suggesting a possible association between antioxidant activity and bacterial inhibition.

The strong correlations between antioxidant activity and bacterial inhibition indicate a statistical association between these parameters; however, these findings do not establish a causal relationship or a shared mechanism of action. The highest correlations were observed with *A. johnsonii*, *A. tumefaciens,* and *B. subtilis*, indicating these strains are most sensitive to the extract’s antioxidant-related mechanisms. These findings align with the known phenomenon in which phenolic and flavonoid compounds contribute to both antioxidant activity and antibacterial effects. These mechanisms may involve the induction of oxidative stress within bacterial cells, disruption of cell membranes, or interference with bacterial metabolism. Gram-negative bacteria, such as *E. coli* and *E. carotovora*, exhibited relatively moderate correlations, likely due to the protective outer membrane, which reduced extract penetration, whereas gram-positive bacteria were more susceptible. Overall, the results suggest that antioxidant-related phytochemicals may contribute to antibacterial activity. This suggests potential applications in natural preservative development, plant protection, or the development of therapeutic agents, where both antioxidant and antimicrobial properties are desirable. Further studies are warranted to isolate the specific antioxidant compounds responsible and determine their precise mechanisms of action against different bacterial strains. Although strong correlations were observed, these findings do not demonstrate causation, and further studies are required to elucidate the mechanisms linking antioxidant compounds to antimicrobial activity.

### 3.6 Correlation heatmap between fungal inhibition and antioxidant activity

**Figure 4:**
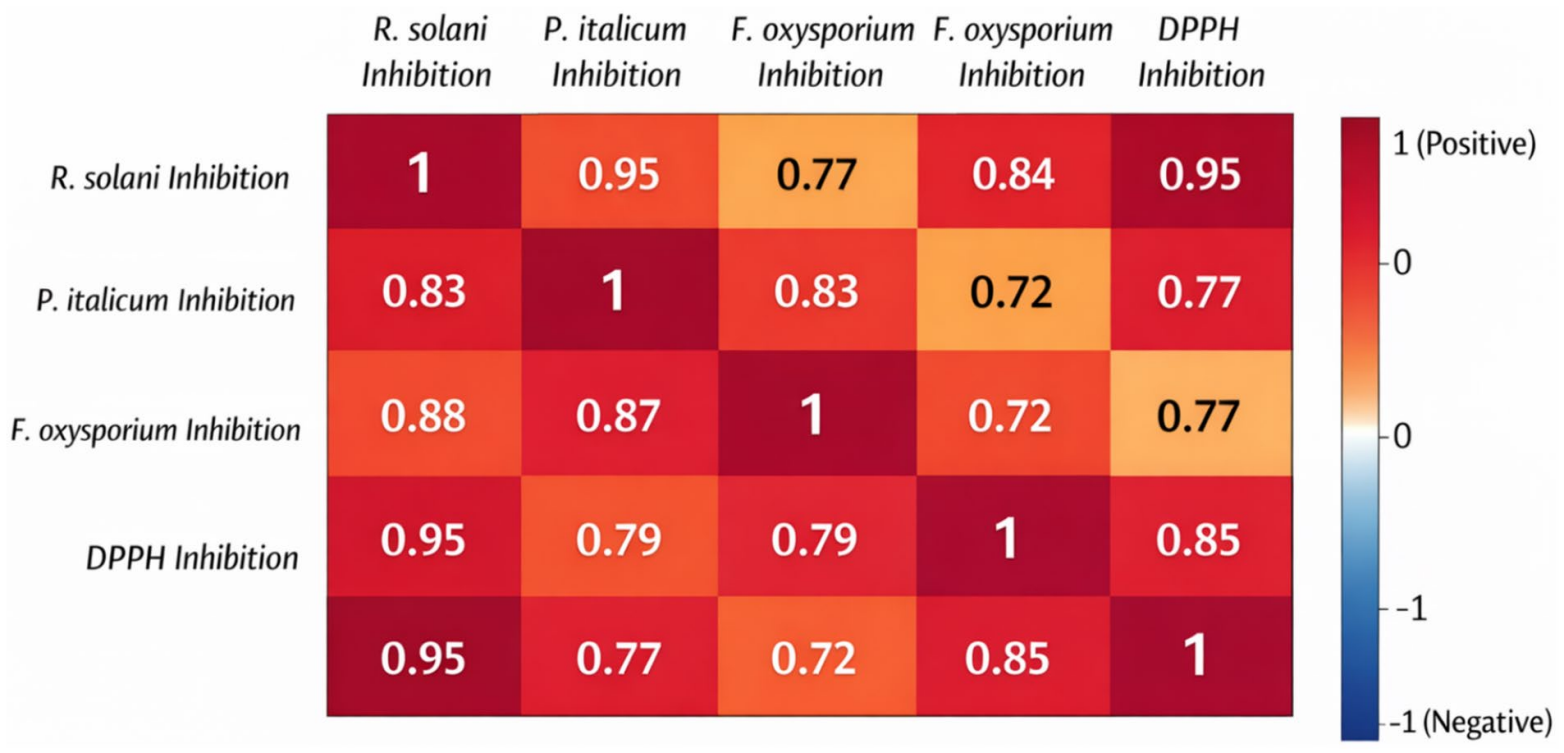
Correlation heatmap between fungal inhibition and antioxidant activity under different extract concentrations.

Pearson’s correlation coefficient was used to assess the association between antioxidant radical-scavenging capacity and mycelial growth suppression by *R. solani*, *P. italicum*, and *F. oxysporum* at varying extract concentrations. The correlation heatmap (Figure 4) revealed strong inverse relationships between antioxidant activity and fungal growth. Antioxidant inhibition showed a strong negative correlation with the mycelial growth of *R. solani* (*r* = − 0.93, *p* < 0.001), indicating that increased antioxidant activity was closely associated with reduced fungal growth. Similarly, *F. oxysporum* showed a strong negative correlation with antioxidant inhibition (*r* = − 0.96), suggesting that this pathogen is highly sensitive to the bioactive components of the extract. In contrast, *P. italicum* showed a moderate negative correlation with antioxidant inhibition (r ≈ −0.58), indicating a comparatively lower susceptibility. Strong positive correlations were observed among fungal growth parameters, reflecting similar growth trends across increasing extract concentrations.

The Pearson correlation heatmap clearly demonstrates a strong correlation between the antioxidant activity and antifungal efficacy of the extract. The strong negative correlations observed for *R. solani* (*r* = −0.93, *p* < 0.001) and *F. oxysporum* (*r* = −0.96) indicate that as antioxidant inhibition increased, mycelial growth decreased markedly. This association may reflect the contribution of shared phytochemical constituents; however, further mechanistic studies are required to confirm this relationship. The comparatively weaker correlation observed for *P. italicum* (*r* = −0.58) suggests that species-specific resistance mechanisms may be in place, including differences in cell wall composition or antioxidant defense systems. Such variability in sensitivity among fungal species has been widely reported in studies using plant extracts as antifungal agents. Overall, the correlation analysis supports the hypothesis that antioxidant capacity is closely linked to antifungal activity, reinforcing the potential application of the extract as a natural antifungal agent.

## 4 Discussion

Preliminary phytochemical screening of *V. nilotica* fruit extract revealed a rich composition of tannins, flavonoids, alkaloids, glycosides, terpenoids, and saponins. This broad spectrum of secondary metabolites underscores the plant’s diverse bioactive potential, providing a biochemical rationale for its extensive ethnomedicinal use. Tannins and flavonoids are particularly notable because they are strongly associated with antioxidant and antimicrobial activities (19). A recent study by Ojo et al. (40) identified high concentrations of total phenolics and flavonoids in the aqueous extracts of *V. nilotica* seeds and pods. The presence of alkaloids and glycosides further supports their potential analgesic, cardioprotective, and neuroprotective properties (21, 41). In contrast, terpenoids and saponins contribute to their anti-inflammatory and immunomodulatory effects (42). Together, these compounds validate the therapeutic versatility of *V. nilotica*.

The methanolic fruit extract demonstrated potent antioxidant activity in the DPPH assay, achieving 87.84% inhibition at 1000 μg mL^−1^ and an IC_50_ of 31.77 μg mL^−1^. Although less potent than ascorbic acid (IC_50_ = 2.88 μg mL^−1^), the extract exhibited a comparable plateau at higher concentrations, indicating a robust radical-scavenging capacity. The observed antioxidant effects can be directly linked to the high total phenolic (419.45 mg GAE g^−1^) and flavonoid (245.48 mg QE g^−1^) contents measured in the extract, as phenolic compounds are known to neutralize free radicals and mitigate oxidative stress (21, 43). Comparable IC_50_ values have been reported for other *Acacia* species, such as *A. confusa* and *A. seyal*, confirming that this genus is a reliable source of polyphenolic antioxidants (44, 45). These results emphasize the potential role of *V. nilotica* as a natural antioxidant, supporting its traditional use in managing oxidative stress-related disorders.

In addition to its antioxidant effects, which protect against oxidative stress, the extract demonstrated antimicrobial potential against a broad spectrum of bacterial and fungal species. Gram-positive strains were generally more sensitive, consistent with the structural differences in cell walls that make gram-negative species more resistant to phytochemicals than gram-positive species. Notably, the extract suppressed fungal pathogens, including *R. solani* and *F. oxysporum*, corroborating previous reports of *V. nilotica’s* antifungal activity (13). This antimicrobial activity can be attributed to the synergistic effects of tannins, flavonoids, and alkaloids present in the extract. Tannins exert antimicrobial effects by disrupting microbial membranes and inactivating enzymes; flavonoids interfere with cell wall synthesis and nucleic acid function; and alkaloids disrupt microbial metabolism (46, 47). Together, these compounds may explain the broad inhibitory spectrum observed and highlight the potential of *V. nilotica* as a source of natural antimicrobial agents, particularly against MDR strains. The minimum bactericidal concentration (MBC) was not determined because of experimental constraints; therefore, we recommend that future studies include broth microdilution assays and MBC determination to strengthen antimicrobial characterization.

In addition to protecting against microbial pathogens, the methanolic extract of *V. nilotica* exhibited concentration-dependent cytotoxicity against MCF-7, PC-3, and Caco-2 cancer cell lines, while maintaining comparatively higher viability in normal Vero cells. Among the tested cell lines, MCF-7 cells were susceptible to both treatments, whereas PC-3 cells showed relative resistance to the plant extract, consistent with the chemoresistance of prostate cancer cells. Caco-2 cells exhibited intermediate responses. The moderate selectivity of *V. nilotica* suggests preferential cytotoxicity toward cancer cells rather than a definitive therapeutic advantage over DXR. This effect is likely mediated by its phytochemical constituents, particularly polyphenols such as tannins and flavonoids, which are known to interfere with tumor growth pathways (19). Specific compounds, such as epigallocatechin-3-O-gallate (EGCG), previously identified in *V. nilotica*, have demonstrated selective anticancer activity by inducing apoptosis in malignant cells while sparing normal cells. However, definitive compound identification was not performed, as advanced chromatographic and spectroscopic techniques (e.g., HPLC-MS/MS, GC–MS, or NMR) are beyond the scope of this study and will be addressed in future work.

Thus, although less potent than DXR, the extract’s selectivity and multi-targeted phytochemical composition make it a promising candidate for complementary or adjuvant cancer therapy (48, 49). Nevertheless, clinical validation is required to confirm its therapeutic advantage over existing agents. Future studies should include mechanistic assays, such as apoptosis detection, ROS quantification, and gene expression profiling, to delineate anticancer pathways.

In summary, these results confirm that *V. nilotica* fruit extract is a rich source of bioactive compounds with potent antioxidant, antimicrobial, and cytotoxic properties. The phytochemical diversity of this plant underlies its broad pharmacological potential, validating its long-standing use in traditional medicine. Broad-spectrum antimicrobial activity was demonstrated, encompassing efficacy against both gram-positive and gram-negative bacteria and fungal pathogens. *In vitro* cytotoxicity assays revealed concentration-dependent anticancer effects that preferentially targeted malignant cells while minimizing toxicity to normal cell populations. Although the extract was less potent than standard drugs, such as ascorbic acid and doxorubicin, its moderate selectivity makes it a potential candidate for further pharmacological investigation.

## Data Availability

The raw data supporting the conclusions of this article will be made available by the authors, without undue reservation.
