# Supplementary material for: Selective anticancer activity of Vachellia nilotica fruit extract: integrated phytochemistry with antioxidant, antimicrobial, and cancer cell targeting
Source: Front Nutr. 2026 Apr 7;13:1799072. doi: 10.3389/fnut.2026.1799072 (PMC13095839; doi:10.3389/fnut.2026.1799072)
Supplement: Supplementary file 1 [file Table_1.DOCX]

**Table S1**. Morphological and physiological characteristics of the isolated *Erwinia* *carotovora* subsp. *atroseptica* (Eca) and *Bacillus subtilis* (Bs)

| Characteristics | Eca | Bs |
| --- | --- | --- |
| Shape of cell  Motility  Gram staining  Oxidase test  Hydrolysis of Casein  Gelatin liquefaction  Hydrolysis of aesculin  Urea test  MR  VP  Nitrate reduction  2-keto gluconate  Starch hydrolysis  Levan production  Catalase test  Indole formation  H_2_S production | Rod  +  -  -  +  +  +  -  -  +  +  +  -  +  +  -  - | Rod  +  +  +  +  +  +  -  -  +  -  +  +  +  +  +  + |

+ = positive reaction - = negative reaction
